# Long-term green-Mediterranean diet may favor fasting morning cortisol stress hormone; the DIRECT-PLUS clinical trial

**DOI:** 10.3389/fendo.2023.1243910

**Published:** 2023-11-14

**Authors:** Liav Alufer, Gal Tsaban, Ehud Rinott, Alon Kaplan, Anat Yaskolka Meir, Hila Zelicha, Uta Ceglarek, Berend Isermann, Matthias Blüher, Michael Stumvoll, Meir J. Stampfer, Iris Shai

**Affiliations:** ^1^Faculty of Health Sciences, Ben-Gurion University of the Negev, Beer-Sheva, Israel; ^2^Department of Medicine, Hebrew University and Hadassah Medical Center, Jerusalem, Israel; ^3^Department of Medicine, University of Leipzig, Leipzig, Germany; ^4^Helmholtz Institute for Metabolic, Obesity and Vascular Research (HI-MAG) of the Helmholtz Zentrum München at the University of Leipzig and University Hospital Leipzig, Leipzig, Germany; ^5^Department of Nutrition, Harvard T.H. Chan School of Public Health, Boston, MA, United States; ^6^Channing Division of Network Medicine, Department of Medicine, Harvard Medical School and Brigham and Women’s Hospital, Boston, MA, United States

**Keywords:** fasting plasma cortisol, lifestyle intervention, mediterranean diet, weight loss, insulin resistance, cardiometabolic health

## Abstract

**Background:**

Fasting morning cortisol (FMC) stress hormone levels, are suggested to reflect increased cardiometabolic risk. Acute response to weight loss diet could elevate FMC. Richer Polyphenols and lower carbohydrates diets could favor FMC levels. We aimed to explore the effect of long-term high polyphenol Mediterranean diet (green-MED) on FMC and its relation to metabolic health.

**Methods:**

We randomized 294 participants into one of three dietary interventions for 18-months: healthy dietary guidelines (HDG), Mediterranean (MED) diet, and Green-MED diet. Both MED diets were similarly hypocaloric and lower in carbohydrates and included walnuts (28 g/day). The high-polyphenols/low-meat Green-MED group further included green tea (3-4 cups/day) and a Wolffia-globosa Mankai plant 1-cup green shakeFMC was obtained between 07:00-07:30AM at baseline, six, and eighteen-months.

**Results:**

Participants (age=51.1years, 88% men) had a mean BMI of 31.3kg/m^2^, FMC=304.07nmol\L, and glycated-hemoglobin-A1c (HbA1c)=5.5%; 11% had type 2 diabetes and 38% were prediabetes. Baseline FMC was higher among men (308.6 ± 90.05nmol\L) than women (269.6± 83.9nmol\L;p=0.02). Higher baseline FMC was directly associated with age, dysglycemia, MRI-assessed visceral adiposity, fasting plasma glucose (FPG), high-sensitivity C-reactive-protein (hsCRP), testosterone, Progesterone and TSH levels (p ≤ 0.05 for all). The 18-month retention was 89%. After 6 months, there were no significant changes in FMC among all intervention groups. However, after 18-months, both MED groups significantly reduced FMC (MED=-1.6%[-21.45 nmol/L]; Green-MED=-1.8%[-26.67 nmol/L]; p<0.05 vs. baseline), as opposed to HDG dieters (+4%[-12 nmol/L], p=0.28 vs. baseline), whereas Green-MED diet FMC change was significant as compared to HDG diet group (p=0.048 multivariable models). Overall, 18-month decrease in FMC levels was associated with favorable changes in FPG, HbA1c, hsCRP, TSH, testosterone and MRI-assessed hepatosteatosis, and with unfavorable changes of HDLc (p<0.05 for all, weight loss adjusted, multivariable models).

**Conclusion:**

Long-term adherence to MED diets, and mainly green-MED/high polyphenols diet, may lower FMC, stress hormone, levels,. Lifestyle-induced FMC decrease may have potential benefits related to cardiometabolic health, irrespective of weight loss.

**Clinical trial registration:**

ClinicalTrials.gov, identifier NCT03020186.

## 1 Introduction

Cortisol is the main glucocorticosteroid (GC) produced by the adrenal glands, specifically in the zona fasciculata of the adrenal cortex and is commonly known as a “stress hormone”. It is a key component of the body’s stress response system, which is regulated by the hypothalamic-pituitary-adrenal (HPA) axis. In addition, cortisol is involved in many physiological processes including metabolism, glycemic control, immune response, growth, cardiovascular function, mood, cognitive functions, reproduction, and development (1). Cortisol levels can exhibit three distinct patterns of fluctuation: ultradian, characterized by pulses of circulating hormone levels occurring approximately every 60 minutes; circadian, representing diurnal fluctuations; and stimulus-induced, triggered by external components that activate the HPA axis. The basal activity often encompasses both the circadian and ultradian rhythms. In humans, cortisol levels reach their peak in the morning and are lowest at night, primarily regulated by the central pacemaker located in the suprachiasmatic nucleus (2).

Dysregulation of cortisol can lead to significant health implications, primarily by inducing insulin resistance and diabetes through its effects on key organs involved in the pathogenesis of insulin resistance, including the liver, skeletal muscle, adipose tissue, and pancreas (3–6). Several studies have reported an association between elevated GCs levels and the development of multiple adverse metabolic complications, including type 2 diabetes, cardiovascular disease, dyslipidemia, and ectopic fat accumulation (3, 4, 7–10). In addition, prolonged exposure to GC’s can lead to the accumulation of central fat, accompanied by low-grade inflammation. Garciía-Eguren et al, identified that, transcriptional and epigenetic signatures induced by elevated cortisol levels in visceral adipose tissue (VAT) may explain the adverse consequences of long-term VAT impairment (11). While research has highlighted the adverse effects of excessive cortisol exposure, it has certain limitations. Much of the research has focused on animal models, while in human studies, the primary emphasis has been on prolonged glucocorticoid therapy and Cushing’s syndrome. the determinants and metabolic consequences of elevated cortisol levels within the physiological ranges in humans are yet to be determined.

Studies of lifestyle interventions on changes in GCs levels have yielded mixed results. In a systematic review conducted by Chawla et al., it was observed that the timing of food consumption, particularly time-restricted eating (TRE), can impact the circadian cortisol fluctuations. Notably, during Ramadan practice of TRE, a reduced waking cortisol response was observed, and a statistically significant increase in cortisol levels in the evening compared to non-Ramadan periods. In non-Ramadan TRE studies, skipping dinner was associated with a decrease in evening cortisol levels and potentially an elevated morning level, although not all cases accounted for waking time (12). Additionally, diet content can. Also alter cortisol fluctuations. Shively et al. demonstrated in non-human primates that adherence to the Mediterranean (MED) diet resulted in reduced cortisol responses to acute stress and ACTH challenges, accompanied by increased stress resilience. Furthermore, Carvalho et al. revealed that the MED diet countered the link between various cortisol biomarkers and inflammation (13, 14). Additionally, specific products rich in polyphenols such as Hibiscus sabdariffa, tea, dark chocolate, as well as specific compounds such as resveratrol and epigallocatechin gallate, have shown associations with cortisol dynamics. This suggests a potential role for these compounds in modulating the body’s response to stress (15–18). Stimson et al., showed that high-fat low-carbohydrate diet enhance cortisol regeneration and reduce cortisol in activation, independent of changes in energy consumption and weight-loss (19).The relationship between caloric restriction and changes in cortisol levels is unclear. Although some studies suggest that acute short-term caloric restriction can increase in cortisol levels, it appears that cortisol elevation is primarily attributable to fasting or a short duration of caloric restriction rather than a long-term caloric restriction (20–22). The limited number of studies and the variability in nutritional interventions make it difficult to draw clear conclusions on the impact of lifestyle interventions on cortisol levels, highlighting the need for further research in this area.

In this study, we aimed to investigate the impact of long-term dietary interventions on fasting morning cortisol (FMC) and its association with metabolic health, beyond the effects of weight loss.

## 2 Methods

### 2.1 Study design

The DIRECT-PLUS trial (ClinicalTrials.gov ID: NCT03020186) was designed as an 18-month study of the effectiveness of a lifestyle intervention program on weight loss and cardiovascular risk reduction. The trial was conducted in an isolated workplace, the Nuclear Research Center Negev (NRCN) in Dimona, Israel, where the participants had access to a monitored lunch programs. Of the 378 volunteers who initially enrolled, 294 met the inclusion criteria of age greater than 30 years with abdominal obesity (waist circumference of over 102cm for men and over 88cm for women) or dyslipidemia (triglycerides greater than 150mg/dL and high-density lipoprotein cholesterol less than or equal to 40mg/dL for men or less than or equal to 50mg/dL for women). The exclusion criteria were an inability to participate in physical activity, abnormal liver function, a serum creatinine level of 2 mg/dL or higher, major illnesses that might require hospitalization, active malignancy or undergoing chemotherapy within the prior 3 years, participation in another trial, treatment with warfarin (due to its interaction with vitamin K), or having an implant that would preclude magnetic resonance imaging (MRI). Furthermore, none of the participants were on continuous steroid or anti-inflammatory treatment. There were no new diagnoses requiring the initiation of anti-inflammatory or steroid therapies during the study. The study protocol was approved by the Soroka University Medical Centre medical ethics board and institutional review board, and all participants provided written informed consent. The participants did not receive any financial compensation or gifts for their participation in the study. Clinical and medical measurements were taken at baseline, 6-months, and 18-months.

### 2.2 Randomization and intervention

Participants were assigned to one of three treatment groups: healthy dietary guidelines (HDG), MED diet, or green MED diet. The randomization process is described in detail in **Supplementary Data 1**. The participants were aware of their assigned intervention (open-label protocol). All participants were provided with a complimentary monitored gym membership and received physical activity instructions. It is crucial to emphasize that this study did not involve a physical activity intervention but rather equal accommodations were provided across all intervention groups. HDG participants were given basic health-promoting dietary recommendations. MED participants were instructed to adhere to a calorie-restricted traditional MED diet, low in simple carbohydrates, as in prior trials (23, 24). The assigned MED diet was high in vegetables, with poultry and fish replacing beef and lamb. MED participants were given 28g of walnuts per day [containing 440 mg polyphenols/day; gallic acid equivalents (GAE), according to United States Department of Agriculture (USDA) Phenol-Explorer: http://phenol-explorer.eu/food-processing/foods, including, mostly, ellagitannins, ellagic acid and its derivatives (25)]. *Green-MED* participants were also given 28g/day walnuts and instructed to avoid red/processed meat. The green MED diet was richer in plants and polyphenols, as participants were specifically provided with 3-4 cups of green tea per day and 400ml green shake of Wolffia globosa (Mankai strain; a newly developed duckweed grown under highly supervised conditions) as a green plant-based protein, replacing animal protein at dinner. Both green tea and Mankai together provided an additional daily intake of 800 mg polyphenols [(GAE), according to Phenol-Explorer and Eurofins lab analysis, including catechins (flavanols)] beyond the polyphenol content in the prescribed MED diet. The calorie count for the day included the green tea and Mankai shake. The MED and green MED diets had the same calorie restriction (1500-1800 kcal/day for men and 1200-1400 kcal/day for women). Further details of the dietary interventions and physical activity protocols are provided in **Supplementry Data 2**.

### 2.3 Nutritional adherence assessment

Self-reported food frequency questionnaires (FFQ) were administered via computer at baseline, after 6 months, and at the trial’s conclusion (26, 27), which encompassed an assessment of provided item intake. We tracked overall changes in specific food group consumption, as previously detailed (28) alongside the intake of Mankai and green tea. The outcomes of these changes have been discussed in our prior work (29).

### 2.4 Lifestyle changes guidance

Regarding lifestyle interventions, participants received 90-minute sessions at the workplace, combining nutritional and physical activity guidance from a multidisciplinary team, including physicians, clinical dietitians, and fitness instructors. All lifestyle educational programs were provided at the same intensity to all three groups, These sessions occurred weekly during the first month, monthly over the next five months, and every other month until the 18th month. Simultaneously, the exercise program involved a gradual increase in aerobic training, starting at 20 minutes and 65% of the maximum heart rate, progressing to 45-60 minutes at 80% of the maximum heart rate, performed 3-4 times per week. Resistance training began with a single set of weights at 60% of the maximum weight and eventually advanced to two sets at 80% of the maximum weight. These resistance exercises included leg extensions, leg curls, squats, lateral pull-downs, push-ups, shoulder presses, elbow flexions, triceps extensions, and bent leg sit-ups. These adjustments aimed to enhance both aerobic and strength components of the workout program. To maintain motivation, participants received timely text messages with relevant information based on their intervention group, and a website provided access to specific nutritional and physical activity information for each assigned intervention group.

### 2.5 Clinical parameters and fasting blood biomarkers

A standard wall-mounted stadiometer was used to measure height to the nearest millimeter. Without shoes, body weight was measured to the nearest 0.1 kg. WC was measured to the nearest millimeter halfway between the last rib and the iliac crest using standard procedures and an anthropometric measuring tape. After resting, two blood pressure (BP) measurements and a pulse rate were recorded using an automatic BP monitor (Accutorr-4, Datascope, New Jersey, USA). The blood pressure was computed as the mean of the 2 measurements. FMC and all other laboratory markers were measured in blood samples drawn between 07:00-07:30 AM after a 12-hour fast. Additionally, participants were instructed to avoid physical activity in the 12 hours preceding their blood tests. We also verified that on the day of each examination, all patients were not currently suffering from acute illnesses and were not receiving acute or subacute medical treatments. All FMC measurements were analyzed by LC-MS/MS in serum, as previously described (30). samples were centrifuged and stored at -80°C. Visceral adipose tissue (VAT) and intrahepatic fat (IHF) were assessed at baseline and after 18-months of intervention by MRI. Further details are provided in **Supplementry Data**.

### 2.6 Statistical analysis

The primary aim of the DIRECT-PLUS randomized controlled trial was to explore the effects of the interventions on weight and adiposity (29, 31).This secondary analysis report explores the dynamics of FMC during weight loss interventions and its associations with metabolic health, beyond weight loss.

Continuous variables are presented as mean ± standard deviation and categorical variables are presented as percentages. Variables were tested for normal distribution using the Shapiro-Wilk test. Baseline characteristics of the study population were analyzed across sex-specific tertiles of baseline FMC; we tested for trend using the kendall tau test. Correlations were evaluated using Pearson’s or Spearman’s correlation tests based on the distribution of the variables (normal vs. non-normal). 5 participants were lacking FMC at baseline and were not included in further analysis (n=289). VAT were expressed as proportions out of abdominal adipose tissue depots assessed (I.e., Deep-subcutaneous fat, superficial-subcutaneous fat and visceral adipose fat); this was to genuinely reflect the abundancy of each fat layer, irrespective of total adipose tissue. IHF was assessed using H-MRS as elaborated in **Supplement 3**. To assess the relationship between changes in FMC by intervention and sex-based groups, we first examined the differences within each group, compare to baseline, using a paired-sample t-test for variables that were normally distributed or the Wilcoxon signed-rank test for variables that were not normally distributed. For assessing between groups differences at baseline across intervention group we used analysis of variance (ANOVA) model. Subsequently, we evaluated between groups differences in FMC dynamics, separately comparing intervention groups and sex-based groups using a multivariable model (Analysis of covariance[ANCOVA]), adjusting for weight loss since baseline, age, and sex for the intervention group comparisons, and weight loss since baseline and age for the sex-based group comparisons. In addition, the change in FMC was presented as mean percentage of change from baseline, absolute change from baseline and median with IQR, due to its high variability. To examine the association between changes in FMC and markers of metabolic health, we conducted partial correlation analyses. Specifically, we examined the relationship beyond the effect associated with weight loss at time 18 using three types of models: a crude (univariate) model, model 1 adjusted for age, sex, and intervention group, and model 2 adjusted for age, sex, intervention group, and weight loss change. The same analyses were performed for changes over 6 months and reported in **Supplementary Figure 1**. All associations in the partial correlation analyses were adjusted for multiple comparisons using FDR-BH (with a q value of 5%(. We examined the differences within glycemic-groups and glycemic-status shifting groups classified by their transition from three glycemic states (Normoglycemic, Pre-diabetic, Diabetic). Glycemic status was defined following the American Diabetes Association (ADA) guidelines (32, 33). Static glycemic migration (SGM) classified as participants who started (Baseline) and finished (18-months) in the same glycemic state (i.e., from normoglycemic to normoglycemic, Pre-diabetic to Pre-diabetic and Diabetic to Diabetic). Negative glycemic migration (NGM) classified as participants who had a worsened state change from baseline to 18-months(i.e., from normoglycemic to pre-diabetic and from pre-diabetic to diabetic). Positive glycemic migration (PGM) classified as participants who improved their glycemic state from baseline to 18 months. No participant migrated from diabetic to normoglycemic nor from normoglycemic to diabetic. These analyses used paired-sample t-tests for variables that were normally distributed or the Wilcoxon signed-rank test for variables that were not normally distributed. To detect differences between these groups, first we used an ANOVA model and then implemented an independent t-test for the baseline comparison. For the comparison of the percentages change we used a multivariable model (ANCOVA) adjusted for weight loss from baseline, intervention group, age, and sex. Analyses were performed Python Software Foundation, version 3.9.13, available at https://www.python.org/. Significance was set at *P* < 0.05.

## 3 Results

### 3.1 Baseline characteristics

Among the 294 study participants (88% men, mean age 51.1) the mean BMI was 31.3kg/m^2^ and mean FMC=304.07nmol\L); 51% were normoglycemic, 38% prediabetic, and 11% had type 2 diabetes. Baseline FMC was higher among men (308.6 ± 90.05 nmol\L) than women 269.6± 83.9 nmol\L, p=0.02). Baseline FMC levels were similar across intervention groups, (HDG= 307.1± 91.5 nmol\L, MED=304± 81.4 nmol\L, Green-MED= 301.1± 97.3 nmol\L; p= 0.87). Baseline FMC was positively associated with age (r=0.11, p=0.05), dysglycemia (p of trend = 0.02), visceral adiposity (r=0.15, p=0.01), glucose (r=0.18, p<0.01), hsCRP (r=0.10, p=0.08), testosterone (r=0.12, p=0.04), progesterone (r=0.22, p<0.01) and TSH (r=0.11, p=0.05). It is important to highlight that fasting glucose, hsCRP, and progesterone exhibited a trend across the second and third tertiles of baseline FMC, aligning with visceral adiposity and dysglycemia, while this was not evident with changes in age, weight, or BMI. Baseline characteristics across sex-specific tertiles of FMC are further detailed in **Table 1**.

**Table 1:** Baseline characteristics of DIRECT PLUS participants across tertiles of fasting morning coristol, n=289.

	Low tertile	Median tertile	top tertile	P of trend	r-correlation
Cortisol, nmol/L	207.69 ( ± 41.64)	300.62 ( ± 27.57)	402.86 ( ± 52.18)	–	–
count	96	96	97	-	-
**Diabetes and Pre-diabetes, %**	41.67	44.79	61.05	0.02*	–
**Age, years**	49.2 ( ± 9.12)	52.02 ( ± 11.22)	52.19 ( ± 10.91)	0.05*	0.11*
Weight, kg	95.08 ( ± 15.95)	93.51 ( ± 12.91)	92.84 ( ± 13.81)	0.32	0.01
BMI, kg/m2	31.26 ( ± 3.78)	31.48 ( ± 3.86)	31.23 ( ± 4.27)	0.65	-0.03
Waist circumference, cm	109.29 ( ± 9.45)	109.94 ( ± 7.65)	110.07 ( ± 10.88)	0.6	0.06
Diastolic-BP, mm Hg	81.1 ( ± 10.03)	80.92 ( ± 9.74)	80.97 ( ± 10.96)	0.85	0.05
Systolic-BP, mm Hg	127.82 ( ± 11.59)	130.8 ( ± 13.34)	130.46 ( ± 14.55)	0.27	0.08
Blood Biomarkers					
**Fasting glucose, mg/dL**	100.99 ( ± 16.77)	99.74 ( ± 14.69)	109.69 ( ± 33.36)	0.02*	0.18*
Insulin, μU/mL	15.36 ( ± 8.1)	14.57 ( ± 7.17)	14.09 ( ± 8.16)	0.15	-0.06
HOMA IR	3.94 ( ± 2.48)	3.67 ( ± 2.1)	3.69 ( ± 2.35)	0.34	-0.02
HbA1c, %	5.44 ( ± 0.58)	5.45 ( ± 0.45)	5.55 ( ± 0.83)	0.8	0.04
Cholesterol, mg/dL	189.55 ( ± 30.85)	191.55 ( ± 35.87)	190.14 ( ± 32.59)	0.89	0.08
Triglycerides, mg/dL^&^	4.93 ( ± 0.41)	4.84 ( ± 0.51)	4.93 ( ± 0.41)	0.8	*0.10*
LDLc, mg/dL	126.46 ( ± 29.47)	124.11 ( ± 30.09)	124.83 ( ± 29.83)	0.55	0.04
HDLc, mg/dL	43.42 ( ± 9.94)	48.36 ( ± 13.08)	46.22 ( ± 11.46)	0.11	0.04
FFA mmol/L	0.49 ( ± 0.19)	0.5 ( ± 0.16)	0.49 ( ± 0.16)	0.49	0.01
ALT, U/L	36.19 ( ± 17.76)	33.29 ( ± 15.18)	35.29 ( ± 17.49)	0.69	0.03
AST, U/L	26.57 ( ± 8.64)	25.01 ( ± 7.38)	25.43 ( ± 7.25)	0.43	0.01
ALKP, mg/dL	74.99 ( ± 18.59)	71.53 ( ± 18.72)	76.15 ( ± 20.39)	0.67	-0.02
**hsCRP^&^, mg/L**	0.92 ( ± 0.95)	0.98 ( ± 0.72)	1.11 ( ± 0.9)	0.04*	*0.10*
IL6 pg/m	3.69 ( ± 1.71)	4.23 ( ± 4.97)	3.89 ( ± 2.31)	0.88	-0.01
Leptin, ng/mL	13.01 ( ± 8.99)	14.2 ( ± 11.26)	15.22 ( ± 13.79)	0.92	-0.07
Fetuin A, μg/mL	334.49 ( ± 95.03)	347.73 ( ± 94.21)	350.24 ( ± 98.92)	0.27	0.09
Chemerin, ng/mL	206.78 ( ± 46.18)	202.26 ( ± 36.4)	215.11 ( ± 47.1)	*0.09*	0.08
FGF 21, pg/mL	193.65 ( ± 119.25)	175.68 ( ± 109.21)	225.49 ( ± 148.95)	0.15	0.09
**TSH mIU/liter**	2.28 ( ± 1.47)	2.33 ( ± 1.05)	2.44 ( ± 1.14)	0.14	0.11*
Folic Acid, ng/dl	7.65 ( ± 3.12)	8.65 ( ± 4.12)	7.85 ( ± 3.4)	0.82	0.04
Ghrelin, pg/mL	527.76 ( ± 235.15)	520.11 ( ± 227.42)	531.45 ( ± 283.47)	0.65	*-0.11*
Aldosterone, nmol/L	231.69 ( ± 182.18)	208.21 ( ± 118.48)	225.7 ( ± 114.07)	0.84	0.04
Progesterone, nmol/L					
**Entire**	2.17 ( ± 8.74)	0.87 ( ± 3.81)	0.36 ( ± 0.16)	>0.01*	0.22*
**Women**	17.6(± 20.64)	2.70(± 8.04)	0.38(± 0.27)	0.11	-0.31
**Men**	0.27( ± 0.07)	0.32( ± 0.19)	0.35( ± 0.13)	>0.01*	0.31*
Testosterone, nmol/L					
**Entire**	13.15 ( ± 6.18)	14.12 ( ± 7.49)	13.92 ( ± 6.7)	0.39	0.12*
**Women**	0.77( ± 0.41)	0.93( ± 0.41)	0.95( ± 0.53)	0.32	0.15
**Men**	14.74( ± 4.54)	15.82( ± 6.12)	15.75( ± 4.88)	0.26	0.05
Estradiol, pmol/L					
**Entire**	123.37 ( ± 107.29)	113.84 ( ± 131.85)	107.02 ( ± 96.75)	*0.07*	*-0.11*
**Women**	309.25(± 213.66)	237.66( ± 356.19)	226.94( ± 239.67)	0.27	-0.18
**Men**	98.90( ± 42.05)	96.53( ± 44.40)	90.82( ± 40.85)	0.17	-0.06
MRI derived fat deposits					
**VAT proportion, %**	28.32 ( ± 7.78)	27.36 ( ± 8.46)	30.57 ( ± 9.97)	0.04*	0.15*
IHF content, %	10.46 ( ± 8.84)	10.38 ( ± 8.63)	9.91 ( ± 8.75)	0.52	-0.04

1. Values are reported as mean ± SD.

2. P of trend was analyzed using Kendall’s Tau test.

3. Sex-specific Tertiles: Q1: men <=265 nmol/L; women: <=232 nmol/L; Q2: men: 266 nmol/L to 351 nmol/L women: 233 nmol/L to 304 nmol/L; Q3: men: =>352 nmol/L, women: =>305 nmol/L.

4. FMC, fasting morning cortisol; BMI, body mass index; BP, blood pressure; HOMA-IR, Homeostatic Model Assessment for Insulin Resistance; HbA1c, glycated-hemoglobin-A1c; LDL,Low-density lipoprotein cholesterol; HDLc, high density lipoprotein cholesterol; FFA, free -fatty acids; ALT, Alanine Transaminase; AST, Aspartate aminotransferase; ALKP, alkaline phosphatase; hsCRP, High Sensitivity C-Reactive Protein; IL6, Interleukin-6; FGF-21, Fibroblast Growth Factor 21; TSH, Thyroid Stimulating Hormone; VAT, visceral adipose tissue; DSC, deep subcutaneous adipose tissue; SSC, superficial subcutaneous adipose tissue; IHF, intrahepatic fat.

5. *p<0.05.

6. italic font style = p<0.1.

7. ^&^Lan transformed.

8. The gonadal hormone levels were calculated for the same tests as presented in the entire table. However, the analysis further stratified these hormone levels by gender, including women and men. Subsequent testing was conducted within each of these strata to examine potential variations.

### 3.2 Adherence to the intervention

As reported previously (29, 31), the retention rate of the study was 98% after 6-months and 89% after 18-months. Dropouts rates were primarily due to a lack of motivation and unrelated medical issues. Moderate weight loss was observed in both calorie-restricted MED groups (MED: -2.81% ± 5.6% [-2.71 ± 5.6 kg]; Green-MED: -3.9% ± 6.3% [-3.7 ± 6.26 kg]), which was significantly greater than that in the HDG group (-0.4% ± 5% [-0.4 ± 4.7 kg]) (p < 0.05 for both MED groups vs. HDG group). The Green-MED diet group exhibited a significant increase in the consumption of fish, Mankai, and green tea, and a decrease in the consumption of red meat and poultry compared to the other two groups (p < 0.01 for all). Furthermore, both the Green-MED and MED diet groups showed a decrease in carbohydrate consumption during both the 18-month and 6-month periods of the trial (p<0.05 for all). Additional information regarding changes in macronutrient intake across intervention groups can be found in **Supplementary Table 2**.

### 3.3 FMC dynamics

**Figure 1:**
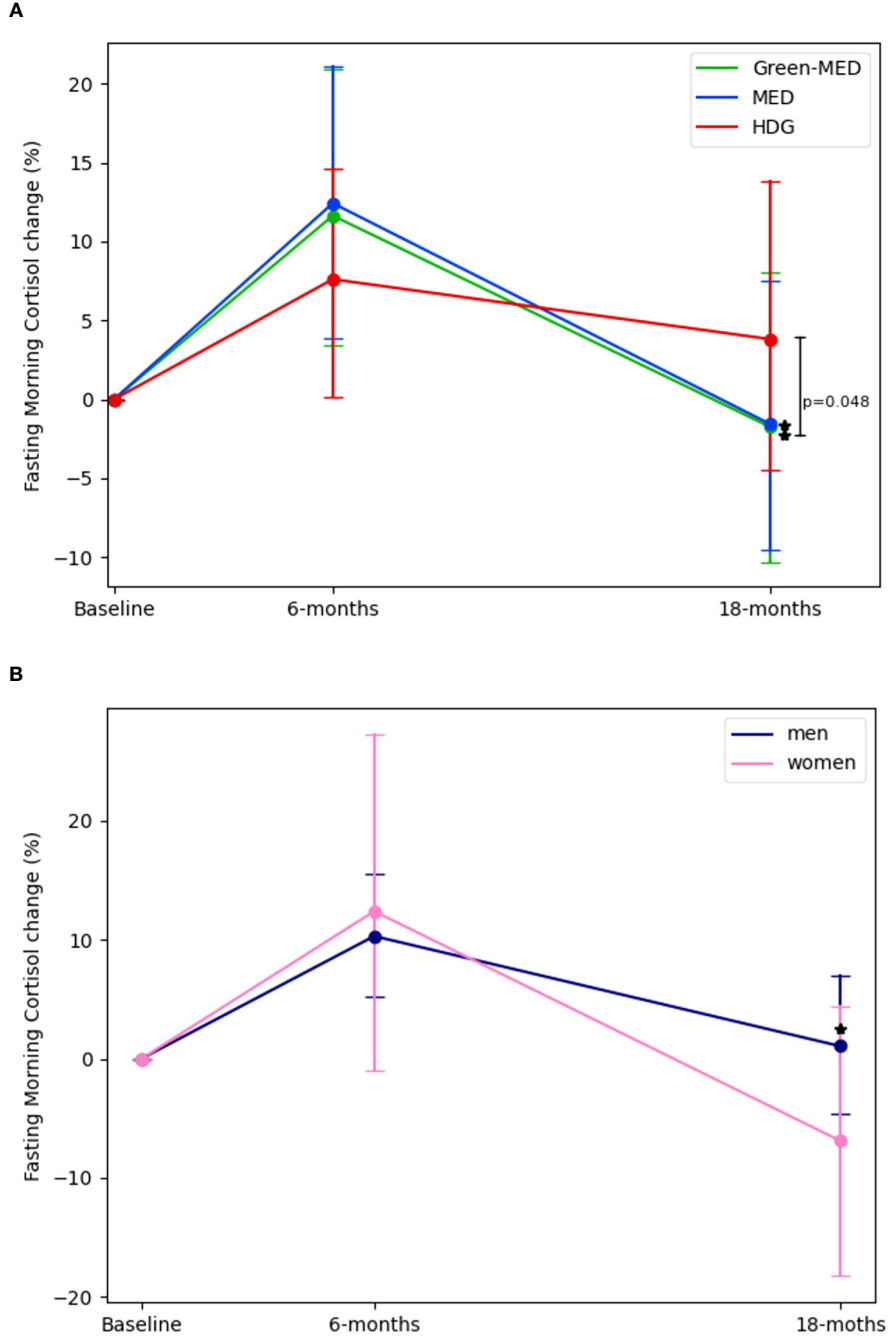
Fasting morning cortisol changes across intervention and sex groups. 1. **(A)**:FMC changes across intervention by time, Baseline, 6-months and 18-months (end of intervention). **(B)**: FMC changes across sex by time, Baseline, 6-months and 18-months (end of intervention) 2. For panel **(A)**. p between groups was analyzed in an ANCOVA model adjusted for Δweight, age, and gender. For panel **(B)**. p between groups was analyzed in an ANCOVA model adjusted for Δweight and age. 3. * significant within change vs. baseline at 0.05; FMC levels in both MED and Green-MED diets reduced significantly vs. baseline. There were no significant differences between sex groups. 4. Abbreviations: HDG, healthy dietary guidelines; Med, Mediterranean diet; Green-Med, green-Mediterranean diet.

At baseline, FMC levels were not statistically different across intervention groups (HDG =307 ± 91.5 nmol/L, MED=304.05 ± 81.4 nmol/L, Green-MED=301.1 ± 97.3 nmol/L; p=0.89). Following a 6-month intervention period (HDG=317.3 ± 126 nmol/L, MED=328.1 ± 107.5 nmol/L, Green-MED=313.5 ± 106.27 nmol/L) (**Figure 1A**), there were no significant changes in FMC among all intervention groups (mean FMC percentages and absolute change: HDG= 7.6%± 38.8% [10.72 ± 114.10 nmol/L], MED=12.4%± 41.5% [22.37 ± 108.7 nmol/L], Green-MED=11.6% ± 44.6% [13.1 ± 111.2 nmol/L]; Median percentages change and IQR: HDG= 3.6%[-20.61%,32.09%], MED=3.1%[-15.49%, 34.34%], Green-MED=3.08%[-17.5%, 29.96%]) as compared to baseline. Similarly, after a 6-month intervention, the levels of FMC in men (323.42 ± 112.22 nmol/L) and women (290.7 ± 119.54 nmol/L) did not show any significant changes within their respective groups (men=10.3% ± 41.6% [14.6 ± 112 nmol/L], women=12.4% ± 42.4% [21.26 ± 106 nmol/L]) (**Figure 1B**).

However, after 18-months(HDG=297.51 ± 85.81 nmol/L, MED=281.82 ± 76.6 nmol/L, Green-MED=273 ± 98.6 nmol/L), both the Med and Green-MED diets achieved significant reductions in FMC levels (mean FMC percentages and absolute change: MED=-1.6% ± 39% [-21.45 ± 92.7 nmol/L], Green-MED=-1.8% ± 45% [-26.67 ± 117.1 nmol/L]; p<0.05 vs. baseline for both; Median percentages change and IQR: MED=-7%[-25.86%, 12.8%], Green-MED=-10.47%[-29.12, 17.83]), while the HDG group showed a non-significant change in FMC (mean FMC percentages and absolute change:+4% ± 46.54% [-12 ± 104 nmol/L], p=0.28; Median percentages change and IQR: HDG=-3.9%[-27.46%,18.9%]). Additionally, after 18-months men (289 ± 86 nmol/L) experienced a significant change in FMC levels (1% ± 44.9% [-19.2 ± 107.9 nmol/L], p<0.01) whereas the women’s group (237.45 ± 83.9 nmol/L) displayed non-significant reduction (-6.9 ± 31.2% [-27.2 ± 80.93 nmol/L], p=0.08). The difference in FMC percentage change between the Green-MED and HDG groups at 18-months, was statistically significant (p=0.048, multivariable model adjusted for age, sex, and weight-loss from baseline). There were no significant differences between sex-based groups at each time point (multivariable model adjusted for age and weight-loss from baseline).

**Figure 2:**
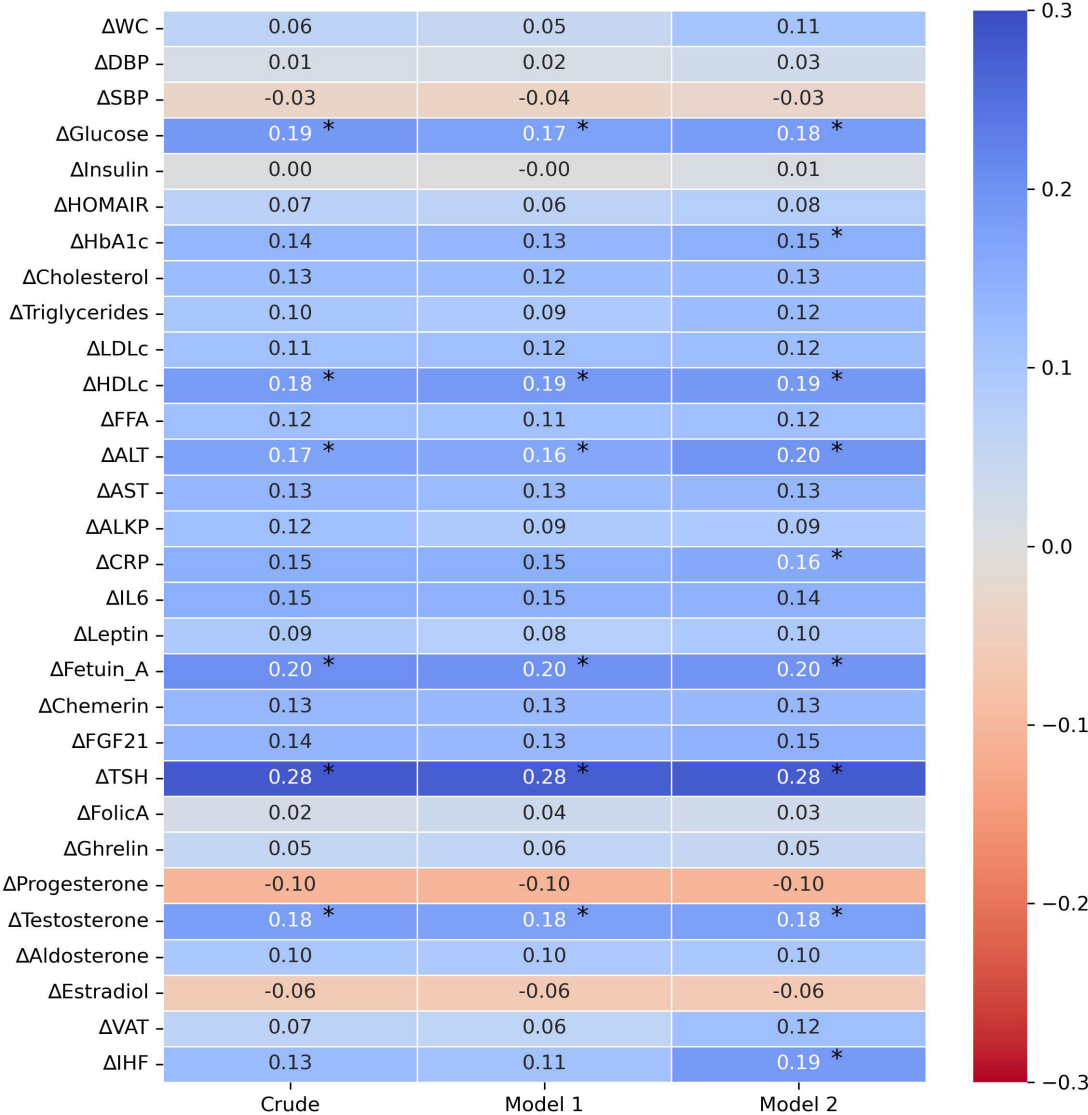
Crude and adjusted correlations between 18-month biomarkers changes and 18-month fasting morning cortisol change. 1. Partial correlation analysis of Δvariable_18months_ with ΔFMC_18months_ 2. Crude – univariate correlation of Δvariable_18months_ with ΔFMC_18months_ 3. Model 1 – adjusted for: age, gender, and intervention group. 4. Model 2 – adjusted for: age, gender, intervention group and Δweight_18months_. 5. Abbreviations: FMC, fasting morning cortisol; BMI, body mass index; BP, blood pressure; HOMA-IR, Homeostatic Model Assessment for Insulin Resistance; HbA1c , glycated-hemoglobin-A1c; LDL ,Low-density lipoprotein cholesterol; HDLc, high density lipoprotein cholesterol; FFA, free -fatty acids; ALT, Alanine Transaminase; AST, Aspartate aminotransferase; ALKP, alkaline phosphatase; hsCRP, High Sensitivity C-Reactive Protein; IL6, Interleukin-6; FGF-21, Fibroblast Growth Factor 21; TSH, Thyroid Stimulating Hormone; VAT, visceral adipose tissue; IHF, intra-hepatic fat. 6. * p≤0.05. 7. All pvalues are corrected for multiple comparisons (FDR-BH, Q=5%).

To further examine the association between the 18-month change in FMC and various metabolic biomarkers, we performed three correlation models. In a univariate correlation model, 18-months FMC decrease was positively correlated with 18-month changes of glucose (r=0.19), ALT (r=0.17), Fetuin A (r=0.20), TSH (r=0.28), and testosterone (r=0.18) and HDLc (r=0.18), p<0.05 for all. After adjusting for age, sex, and intervention group, the same markers remained significantly correlated with the 18-month change in FMC (glucose=0.17, HDLc=0.19, ALT=0.16, Fetuin A= 0.2, TSH=0.28, testosterone=0.2). Additionally, after further adjustment for 18-month weight loss, the 18-month decrease in FMC levels was positively correlated with 18-month changes of glucose (r=0.18), HbA1c (r=0.15), ALT (r=0.2), hsCRP (r=0.16), Fetuin A (r=0.2), TSH (r=0.28), MRI-assessed IHF (r=0.19), testosterone (r=0.18) and HDLc (r=0.19), p<0.05 for all. Further associations are illustrated in **Figure 2**. Associations of 6-months change FMC and 6-months change of metabolic markers is illustrated in **Supplementary Figure 1**.

### 3.4 Cortisol dynamic and glycemic status

**Figure 3:**
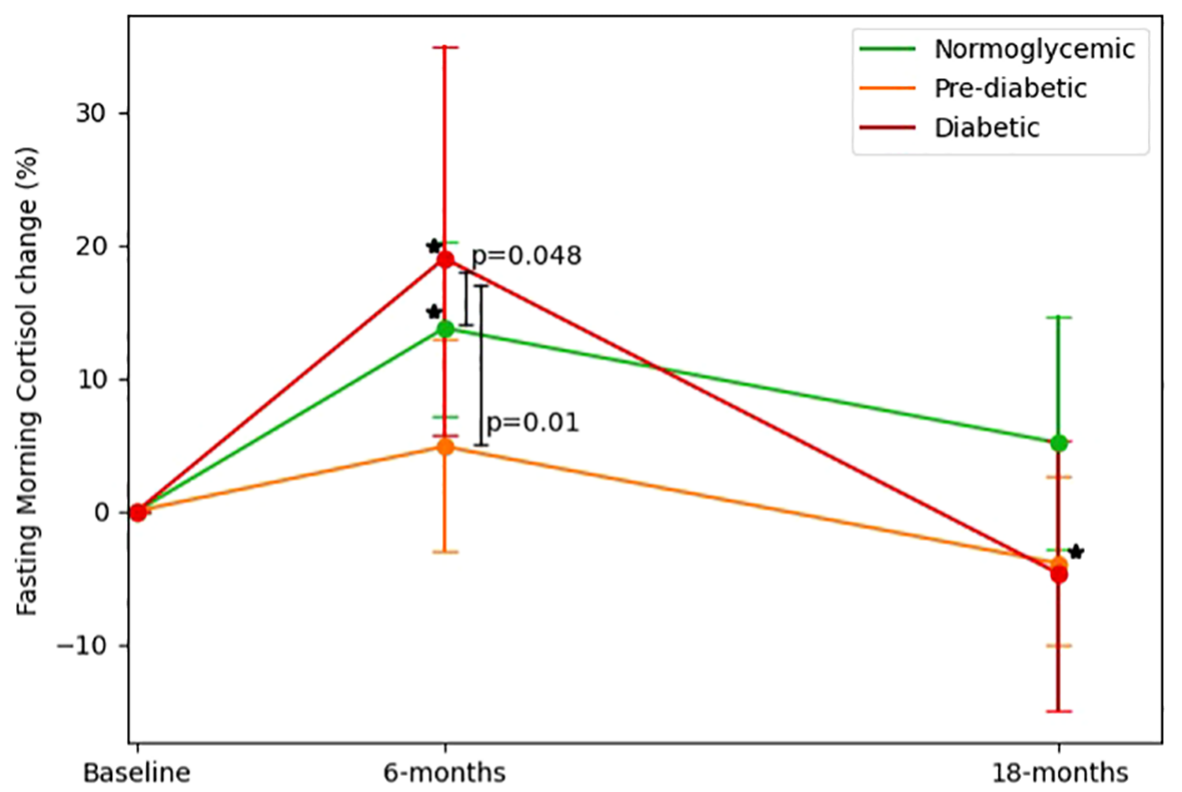
Fasting morning cortisol changes across glycemic-status groups. 1. FMC changes across glycemic status groups (defined at baseline) by time, Baseline, 6-months and 18 months (end of intervention). 2. Diabetic defined as fasting glucose >=126 or HbA1c >=6.5 or receiving medical treatment for diabetes. 3. Pre-diabetic defined as 126>fasting glucose >=100 or 6.5>HbA1c>=5.7. 4. P between groups was analyzed in an ANCOVA model adjusted for Δweight, age, and gender. 5. * Significant (p<0.05) within change vs. baseline at 0.05; Diabetic and normoglycemic groups had significantly elevated FMC levels in 6-months and pre-diabetic groups had significantly reduced FMC levels.

We further examined FMC changes from three glycemic states at baseline: Normoglycemic(Baseline FMC=285.44 ± 85.44 nmol/L), Pre-diabetic (Baseline FMC=320.65 ± 90.52 nmol/L), Diabetic (Baseline FMC=325.65 ± 92.5 nmol/L). At baseline diabetic and pre-diabetic groups had higher FMC levels than the normoglycemic group(p<0.05 for both). After 6-months (Normoglycemic=308.28 ± 106.44 nmol/L, pre-diabetic=319.28 ± 112.04 nmol/L, diabetic=370.8 ± 134.3 nmol/L), the group of probands with type 2 diabetes showed the greatest elevation (19%± 42.9% [50.37 ± 114.40 nmol/L], p=0.01 vs. baseline), as compared to the pre-diabetic (4.8% ± 42% [-0.70 ± 112.7 nmol/L], p=0.94 vs. baseline) and normoglycemic (13.7%± 40.6% [22.56 ± 105.15 nmol/L], p=0.02 vs. baseline) groups (p<0.05 for difference between groups; in multivariable models adjusted for age, sex, and 6-month weight loss). After 18-months (Normoglycemic=276.32 ± 88.38, pre-diabetic=291.8 ± 86.56 nmol/L, diabetic=299.42 ± 88.41 nmol/L), only the pre-diabetic group exhibited a statistically significant reduction in FMC levels vs. baseline (-3.9% ± 33.6% [-28.9 ± 100 nmol/L]; p<0.01), whereas the diabetic and normoglycemic groups showed a non-significant change vs. baseline (-4.7% ± 29%[-30.12 ± 117.6 nmol/L]; p=0.17; (5.1%± 51.7% [-8.13 ± 102.77 nmol/L]; p=0.36; respectively). Further details are illustrated in **Figure 3**.

**Figure 4:**
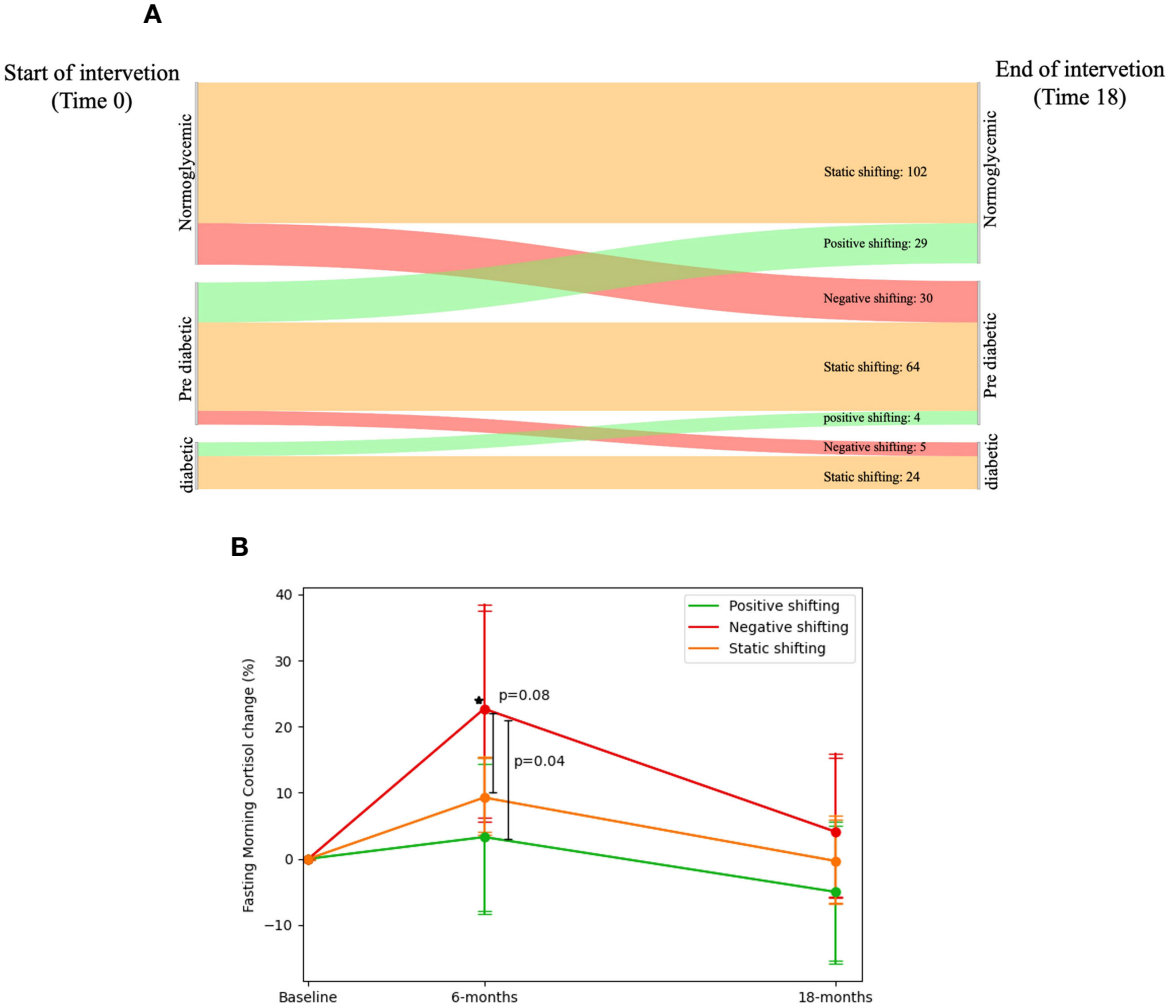
Fasting morning cortisol and shifting of glycemic-status. 1. **(A)** Sankey diagram showing the glycemic-status shifting between three groups: Normoglycemic, Pre-diabetic and Diabetic; 190 participants had a static glycemic-status shift (started and finished in the same group: Normoglycemic → Normoglycemic = 102, Pre-diabetic → Pre-diabetic = 64 and Diabetic → Diabetic = 24), 35 participants who had negative glycemicstatus shifting (started in one group and finished in a worse-diagnosed group: Normoglycemic → Pre-diabetic = 30 and Pre-diabetic → Diabetic = 5), and 33 participants who had a positive glycemic-status shifting (started at one group and finished at a better-diagnosed group: Pre-diabetic → Normoglycemic = 29 and Diabetic → Pre-diabetic = 4). 2. **(B)** FMC dynamics in percent across glycemic-status shifting groups. Between P values analyzed in ANCOVA model adjusted for age, gender, Intervention group and Dweight. 3. Diabetic defined as fasting glucose >=126 or HbA1c >=6.5 or receiving medical treatment for diabetes. 4. Pre-diabetic defined as 126>fasting glucose >=100 or 6.5>HbA1c>=5.7. 5. * significant within change vs. baseline at 0.05; Negative shifting group had elevated FMC levels in 6-months vs. Baseline.

Participants were classified into three groups by their transition from three glycemic status groups [Normoglycemic[n=148, mean weight=91.11 ± 13.01 kg, fasting glucose=91.94 ± 5.55 mg/dL, HbA1c=5.19% ± 0.31%], Pre-diabetic[n=110, mean weight=96.13 ± 16.14 kg, fasting glucose=106.51 ± 7.34 mg/dL, HbA1c=5.51% ± 0.34%], Diabetic[n=32, mean weight=96.36 ± 12.41 kg, fasting glucose=147.16 ± 46.73 mg/dL, HbA1c=6.75% ± 0.97%]; glycemic status groups were defend using ADA definition (32, 33)]. We identified 190 participants who had a static glycemic migration (SGM; started [Baseline] and finished [18-months] at the same group), 35 participants who had negative glycemic migration (NGM; started [Baseline] at one group and finished [18-months] at worse diagnosed group) and 33 participants who had a positive glycemic migration (PGM; started [Baseline] at one group and completed [18-month] at a better diagnosed group). At baseline, NGM group (274.26 ± 70 nmol/L) had lower FMC levels than the SGM (305.14 ± 87.36 nmol/L, p=0.05) and PGM (318.8 ± 110.77 nmol/L, p=0.05) groups. After 6-months(SGM=317.5 ± 107.61, PGM=316 ± 127.85, NGM=326.11 ± 134.26 nmol/L), only the NGM group showed a significant increase in FMC levels (22.71± 49.89% [50.4 ± 128 nmol/L]; p=0.03 vs. baseline) while SGM and PGM showed non-significant elevation in 6-months FMC (9.28% ± 40.87% [12.31 ± 107.62 nmol/L], p=0.12 vs baseline; 3.30%± 35.05% [-2.80 ± 104.88 nmol/L], p=0.87 vs baseline; respectively), 6-months FMC change levels of the NGM group was significantly higher than that of the PGM group, after controlling for age, sex, weight loss at 6-months, and type of intervention (p=0.04). Further details are illustrated in **Figure 4**.

## 4 Discussion

In this trial, we examined the effect of MED diets on fasting morning cortisol levels, and their associations with cardiometabolic markers. Our findings suggest that long-term lifestyle-induced FMC decrease may play a role in metabolic health, independent of weight loss. Additionally, our study highlights the potential of MED diets and green-MED in particular, to effectively lower the stress hormone FMC over the long term.

This study has several limitations. First, the study population was mainly men, reflecting the sex profile of the workplace. While this was taken into account in various models, it may limit the generalizability of the results to women. In addition, caution is needed in interpreting the results related to gender differences. Second, we could not study effects on peak cortisol response, since the blood samples from our participants were collected between 7:00-7:30 AM making it hard to confirm the precise timing of the peak cortisol response. Instead, we relied on a standardized fasting morning cortisol level. Additionally, we were unable to factor in potential sleep alterations, a potentially significant influence on HPA axis regulation (34). Nonetheless, we did analyze the proportion of shift-workers within each diet intervention group, a factor that can potentially affect cortisol secretion. Notably, The proportion of shift-workers did not differ across diet groups neither in the baseline nor at the end of the trial. Future research should consider the potential impact of sleep alterations on FMC. Third, adherence to the interventions was mainly assessed through self-reporting, which may introduce bias and limit the accuracy of the results. However, objective measures such as serum folate analysis, reflecting green leafy vegetable consumption, or urine polyphenols, demonstrated the accuracy of the reporting (26, 29, 35). In addition, given the subtle differences in FMC and the almost borderline statistical significance of the findings, further research is needed to validate our results. Lastly, it is important to note that we relied solely on self-reported data and did not employ objective measures for physical activity assessment. While physical activity might influence FMC levels, it is essential to underscore that that our study did not involve a physical activity intervention; instead, we provided equivalent accommodations across each intervention group.

This trial has several noteworthy strengths. One key strength is that the study was conducted in a closed workplace setting, providing participants with access to an on-site clinic and a monitored provided lunch. This facilitated intensive dietary guidance and group meetings with multidisciplinary teams of healthcare professionals including physicians, dietitians, and physical trainers, ensuring high adherence. The relatively large sample size and high retention rate enhanced the statistical power and strengthened the reliability of the results.

Cortisol levels may be influenced by various lifestyle interventions such as timing of food intake (12), dietary restriction (20–22), and macronutrient content (19). Despite the potential influence of diet on cortisol levels, the association between the MED diet and cortisol levels has not been extensively studied. While existing research in this area has produced inconclusive results, some evidence suggests that the MED diet may actually help to reduce cortisol levels (13, 14). This study confirms and considerably extends those findings by demonstrating a reduction in FMC by MED diet in a randomized trial and indicating that the reduction in FMC was associated with a reduction in metabolic health biomarkers including glucose, HbA1c, ALT, hsCRP, Fetuin A, TSH, testosterone, and intra-hepatic fat content, suggesting potential improvement in specific metabolic biomarkers. Interestingly, these biomarkers showed an improvement after accounting for weight loss, suggesting the possibility that FMC reduction may improve metabolic health.

Another notable lifestyle factor we observed was calorie restriction. All participants across the study arms were motivated to achieve weight loss by adhering to their respective diets and engaging in physical activity. As previously reported by our research group (31), the 6-month timepoint was marked by rapid weight loss. This rapid weight loss could potentially trigger a significant stress response from the body. This, in turn, leads us to consider that this highly stressed state of rapid weight loss might be associated with an elevated FMC response as witnessed in our study. However, it is important to highlight that the 6-months changes in FMC across the intervention group, were highly variable. Thus, we also reported a median and IQR for better interpreting the results.

Several studies have investigated the association between elevated cortisol levels and dyslipidemia (36, 37), but the relationship between cortisol and HDL remains inconclusive. The precise contribution of cortisol to HDL levels remains unclear (38, 39). To the best of our knowledge, this is the first study to demonstrate a distinct pattern of FMC reduction resulting from specific dietary interventions, with the observed changes in FMC being independently associated with improved metabolic health, irrespective of changes in weight.

Observational studies have established the relationship of elevated cortisol levels and diabetes risk (4, 10). This is thought to occur through the dysregulation of glucose metabolism by cortisol, leading to insulin resistance and impaired glucose tolerance (8). In this study, We found that participants with diabetes had an elevated cortisol response at 6-months, and those who experienced a worsening shift in glycemic state over the 18-month study period also had an elevated cortisol response at 6-months. These findings suggest that acute changes in cortisol levels may be associated with long-term shifts in glycemic status. Further research is needed to fully understand the underlying mechanisms linking cortisol acute response and diabetes risk.

In conclusion, our study adds to the growing body of evidence on the potential benefits of the MED diet for metabolic health beyond weight loss. The results of our trial suggests that long term adherence to a Green-MED/high polyphenols diet may primarily reduce FMC in obese individuals, potentially resulting in improvements in metabolic health biomarkers. Further research is essential to confirm and establish these preliminary findings, as well as to investigate the long-term effects of these dietary interventions on cortisol levels and metabolic health. This future research should specifically explore the associations between changes in FMC and distinct metabolic biomarkers, nonetheless, our study highlights the importance of considering the role of cortisol in metabolic health.

## Data availability statement

Data may be accessible upon request, subject to approval by the study's principal investigator, IS.

## Ethics statement

The studies involving humans were approved by Soroka University Medical Centre medical ethics board and institutional review board. The studies were conducted in accordance with the local legislation and institutional requirements. The participants provided their written informed consent to participate in this study. This study was conducted in accordance with the CONSORT statement.

## Author contributions

IS, GT, AM, HZ, AK, and ER designed the research and conducted the study. IS, LA, and GT analyzed the data, preformed the statistical analysis, wrote the manuscript, and are responsible for final content. IS, GT, and LA conceived and designed the research. MB, UC, BI and MStu reviewed and edited the manuscript. All authors had full access to all the data in the study and take responsibility for the integrity of the data and the accuracy of the data analysis as well as for the decision to submit the manuscript for publication. IS, GT, and LA are the guarantors of this work and take full responsibility for the work as a whole, including the study design, access to data, and the decision to submit and publish the manuscript. All authors contributed to the article and approved the submitted version.
