# Supplementary material for: Long-term green-Mediterranean diet may favor fasting morning cortisol stress hormone; the DIRECT-PLUS clinical trial
Source: Front Endocrinol (Lausanne). 2023 Nov 14;14:1243910. doi: 10.3389/fendo.2023.1243910 (PMC10682947; doi:10.3389/fendo.2023.1243910)
Supplement: Supplementary file 1 [file DataSheet_1.docx]

Supplementary Material

**The effect of Mediterranean diets on fasting morning cortisol and its relation to cardiometabolic health; The DIRECT-PLUS clinical trial**

**Liav Alufer†^1^, Gal Tsaban†^1^, Ehud Rinott^2^, Alon Kaplan^1^, Anat Yaskolka Meir^1^, Hila Zelicha^1^, Uta Ceglarek^3^, Berend Isermann^3^, Matthias Blüher^3,4^, Michael Stumvoll^3,4^, Meir J. Stampfer^5,6^, Iris Shai^1,5^.**

†These authors contributed equally to this work and share first authorship

^1^Faculty of Health Sciences, Ben-Gurion University of the Negev, Beer-Sheva, Israel;

^2^Department of Medicine, Hebrew University and Hadassah Medical Center, Jerusalem, Israel
^3^Department of Medicine, University of Leipzig, Germany
^4^Helmholtz Institute for Metabolic, Obesity and Vascular Research (HI-MAG) of the Helmholtz Zentrum München at the University of Leipzig and University Hospital Leipzig, Germany
^5^Harvard T.H. Chan School of Public Health, Boston, Massachusetts
^6^Channing Division of Network Medicine, Department of Medicine, Harvard Medical School and Brigham and Women’s Hospital, Boston, Massachusetts.

**Correspondence**:
Iris Shai, RD, PhD, Department of Epidemiology, biostatistics and health sciences in the community, Faculty of Health Sciences, Ben-Gurion University of the Negev, PO Box 653, Beer-Sheva 84105, Israel. E-mail: irish@bgu.ac.il; fax: (972)-8-647-7637/8.

# Supplementary Data

# Supplementary data 1: Randomization protocol and allocation sequence: Recruitment to the study was performed by AYM, HZ, ER, AK, and GT. All eligible participants who signed consent to participate in the trial and completed the baseline measurements were randomized into one of the three intervention groups (HDG, MED, green-MED) in a 1:1:1 ratio and within strata of gender and work status (to ensure equal workplace-related lifestyle features between groups). Randomization was conducted in a single phase using an ad hoc R-based procedure. ER, GT, and AYM performed the randomization process. Participants were notified of their assigned intervention group by a cellular phone text message sent by their group instructor.

# Supplementry methods 2: Dietary and PA interventions outlines.


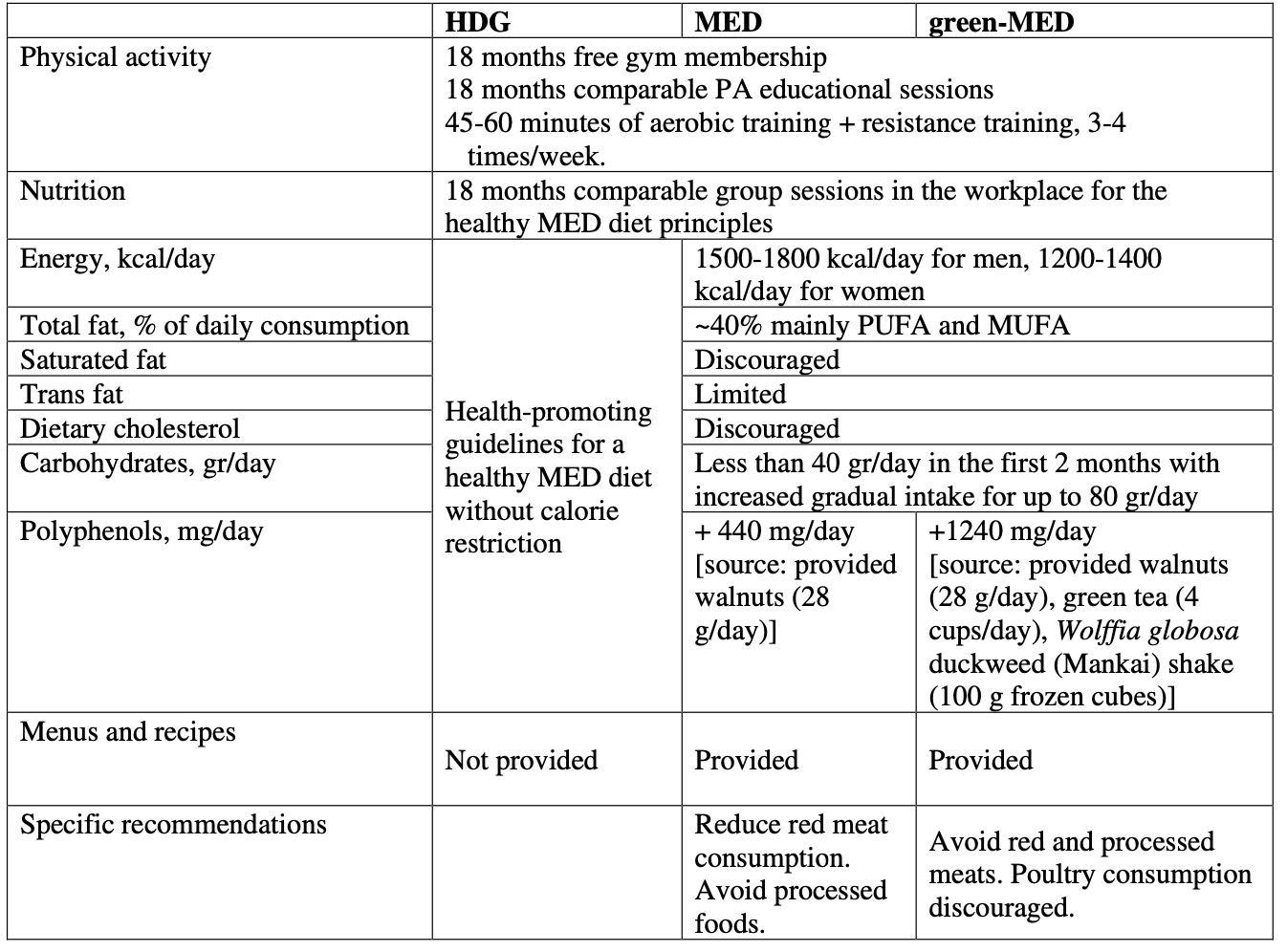


The lifestyle interventions included 90-minute nutritional and PA sessions in the workplace with multidisciplinary guidance (i.e., physicians, clinical dietitians, and fitness instructors). These sessions were held every week during the first month, and then once a month over the following five months. All the lifestyle educational programs were provided at the same intensity to all three groups. To keep the participants motivated, text messages with relevant information for each assigned intervention group were sent on fixed time intervals. In addition, a website listing all nutritional and PA information needed by the participants to continue with the intervention was constructed and was made accessible to the participants according to their intervention group.

- 1. **Supplementry methods 3: Laboratory and Fat deposits assessment methodology:**

Serum total cholesterol (TC;Coefficient-of-variation (CV), 1.3%), HDL-c, low-density-lipoprotein-cholesterol (LDL-c), and TG (CV, 2.1%) were determined enzymatically with a Cobas-6000 automatic analyzer (Roche). Plasma levels of high-sensitivity C-reactive protein (hsCRP) were measured by ELISA (DiaMed;CV, 1.9%). Plasma glucose levels were measured by Roche GLUC3 (hexokinase method). Plasma leptin levels were assessed by ELISA (Mediagnost, CV, 2.4%). Plasma insulin levels were measured with an enzyme immunometric assay (Immulite automated analyzer, Diagnostic Products; CV, 2.5%). The homeostatic model of insulin resistance (HOMA IR) was calculated as follows: insulin(μIU/ml)×glucose(mg/dl)/405^1^. Metabolic syndrome criteria were assessed based on the National Cholesterol Education Program Adult Treatment Panel III criteria^2^. The ten year Framingham risk score (10yFRS) was calculated based on gender, age, total cholesterol and HDL-c levels, systolic-BP and antihypertensive-drug dependence, smoking-status, and diabetes^3^ .All biochemical analyses were performed at the University of Leipzig, Germany. Abdominal fats: were assessed at baseline and after 18 months using 3-Tesla MRI (Ingenia 3.0 T, Philips Healthcare, Best, the Netherlands) scans ^4^. The scanner utilized a 3D modified DIXON (mDIXON) imaging technique without gaps (2mm thickness and 2mm of spacing), fast-low-angle shot (FLASH) sequence with a multi-echo two excitation pulse sequence for phase-sensitive encoding of fat and water signals (TR,3.6ms; TE1,1.19ms; TE2,2.3ms; FOV 520×440×80mm; 2×1.4×1mm voxel size). Four images of the phantoms were generated, including in-phase, out-phase, fat, and water phase ^5^. A breath-hold technique was used to avoid motion artifacts when the abdomen was scanned. The quantifications were performed blindly to the intervention groups. The Inter-class reliability comparing a previous trial^6^ and Intra-class reliability correlations were >0.96 (P<0.001). We quantified abdominal fat using the MATLAB-based semi-automatic software ^4^. We drew a continuous line over the fascia superficialis to differentiate between the deep-SAT and superficial-SAT, and calculated mean VAT, deep-SAT and superficial-SAT from two axial slices: L5-S1 and L4-L5. Quantification of the fat mass regions included the area of each fat type and its proportion (percentage) of the total area of all three abdominal fat types. Intrahepatic fat (IHF): was assessed at baseline and after 18-months using H-MRS ^5^. Localized, single-voxel proton spectra were acquired using a 3.0T magnetic resonance scanner (Philips Ingenia, Best, The Netherlands). The measurements were taken from the right frontal lobe of the liver, with a location determined individually for each subject using a surface, receive-only phased-array coil. Spectra with and without water suppression were acquired using the single-voxel stimulated echo acquisition mode (STEAM) with the following parameters: TR=4000msec, TE=9.0msec, and TM=16.0msec. The receiver bandwidth was 2000Hz, and the number of data points was 1024. Second-order shimming was used. Four averages were taken in a single breath hold for an acquisition time of 16 sec. The voxel size varied somewhat according to anatomy but was approximately 50(AP) × 45(RL) × 54(FH) mm. Water suppression was achieved using the MOIST (Multiple Optimizations Insensitive Suppression Train) sequence consisting of four phase-modulated T1 and B1 insensitive pulses with a 50Hz window. Data analyzed using Mnova software (Mestrelab Research, Santiago de Compostela, Spain) by an experienced physicist blinded to the intervention groups, who also performed visual quality control of fitted spectra. The total hepatic fat fraction in the image was determined as the ratio between the sum of the area under all fat divided by the sum of area under all fat and water peaks ^5^.

# Supplementary Figures and Tables

## Supplementary figure 1. Crude and adjusted correlations between 6-month biomarkers changes and 6-month fasting morning cortisol change.


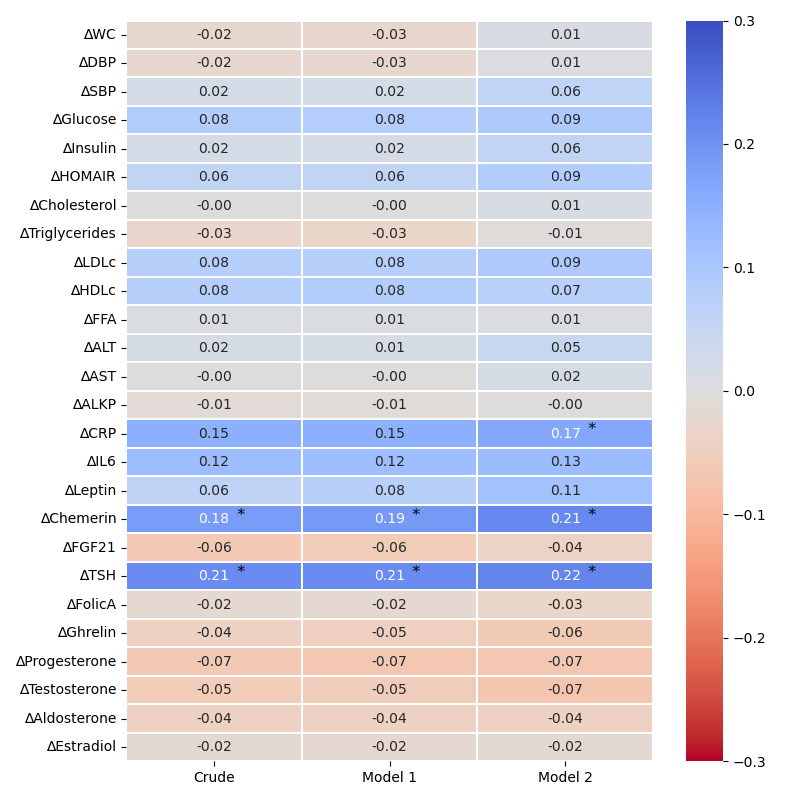


- Crude – univariate correlation of ∆variable_6months_ with ∆FMC_6months_
- Model 1 – adjusted for: age, gender, and intervention group.
- Model 2 – adjusted for: age, gender, intervention group and ∆weight_6months_.
- Abbreviations: FMC, fasting morning cortisol; BMI, body mass index; BP, blood pressure; HOMA-IR, Homeostatic Model Assessment for Insulin Resistance; HbA1c , glycated-hemoglobin-A1c; LDL ,Low-density lipoprotein cholesterol; HDLc, high density lipoprotein cholesterol; FFA, free -fatty acids; ALT, Alanine Transaminase; AST, Aspartate aminotransferase; ALKP, alkaline phosphatase; hsCRP, High Sensitivity C-Reactive Protein; IL6, Interleukin-6; FGF-21, Fibroblast Growth Factor 21; TSH, Thyroid Stimulating Hormone.
- * = p<=0.05
- Diabetic defined as fasting glucose >=126 or HbA1c >=6.5 or receiving medical treatment for diabetes
- Pre-diabetic defined as 126>fasting glucose >=100 or 6.5>HbA1c>=5.7
- ∆FMC is in %

**2.2. Supplementary table 1. Baseline lifestyle and Pharmacotherapy characteristics.**

|  | HDG | MED | Green-MED | All | P between groups† |
| --- | --- | --- | --- | --- | --- |
| Smoking, % | 19.4 | 13.3 | 16.3 | 16.3 | 0.51 |
| Shift workers, % | 22.4 | 23.4 | 23.4 | 23.1 | 0.98 |
| Diabetes, % | 10.4 | 9.2 | 13.4 | 11.0 | 0.63 |
| **Pharmacotherapy** |  |  |  |  |  |
| Anti-hypertensive, % | 14.3 | 11.2 | 16.3 | 13.9 | 0.59 |
| Cholesterol lowering, % | 11.2 | 8.2 | 14.3 | 11.2 | 0.40 |
| Anti-platelet, % | 7.1 | 3.1 | 9.2 | 6.5 | 0.21 |
| Exogenous insulin, % | 1.0 | 1.0 | 3.1 | 1.7 | 0.27 |

†according to the chi-square test. HDG, healthy dietary guidelines; MED, Mediterranean.

**2.3. Supplementary table 2. Baseline 6-months and 18-month changes in reported dietary intake across intervention groups.**

|  | **HDG** | **MED** | **Green-MED** | **p between groups** |
| --- | --- | --- | --- | --- |
| **Macronutrients** |  |  |  |  |
| **Energy** |  |  |  |  |
| Energy at baseline (kcal/day) | 2193±1180.7 | 2200.2±1119.3 | 2065.9±955.8 | 0.63 |
| Energy change from baseline to 6-months (kcal/day) | −601.0±1365.7 | −664.3±917.8 | −724±781.65 | 0.777 |
| change from baseline to 6-months, % | −20.8±47.1 | −21.8±52.5 | −28.5±26.7 | 0.503 |
| Energy change from baseline to 18-months (kcal/day) | -336±1046 | -666±1021 | -544±975 | 0.73 |
| change from baseline to 18-months, % | -11.6±42.9 | -23±27.6 | -20±32 | 0.17 |
| **Total carbohydrates** |  |  |  |  |
| % of energy at baseline | 45.3±7.0 | 44.4±8.5 | 46.3±7.6 | 0.24 |
| % change out of total energy intake at 6 months | −2.9±8.8 | −10.6±9.4 | −8.2±10.6 | **<0.001** |
| Change in g/d from baseline to 18-months, % | -14.0±36.4 | -29.8±31.3 | -27.9±34.0 | **0.003** |
| **Protein** |  |  |  |  |
| % of energy at baseline | 20.6±4 | 20.9±4.6 | 19.9±3.9 | 0.29 |
| % change out of total energy intake at 6-months | 1.0±5.1 | 4.7±6.0 | 4.4+5.1 | **<0.001** |
| % change out of total energy intake at 18-months | -5.8±58.3 | -12.8±38.4 | -14.3±40.2 | 0.6 |
| **Total fat** |  |  |  |  |
| % of energy at baseline | 34.5±4.5 | 35.2±4.7 | 34.5±5.23 | 0.45 |
| % change out of total energy intake at 6-months | 2.1±5.8 | 6.1±5.8 | 5.3±7.3 | <0.001 |
| Change in g/d from baseline to 18-months, % | -7.1±58.5 | -15.7±32.0 | -10.9±38.4 | 0.84 |

Data are means ± Standard deviations for continuous parameters and percentage for categorical parameters.

1. Matthews, D. R. *et al.* Homeostasis model assessment: insulin resistance and beta-cell function from fasting plasma glucose and insulin concentrations in man. *Diabetologia* **28**, 412–419 (1985).

2. Huang, P. L. A comprehensive definition for metabolic syndrome. *Dis Model Mech* **2**, 231–237 (2009).

3. D’Agostino, R. B. *et al.* General cardiovascular risk profile for use in primary care: the Framingham Heart Study. *Circulation* **117**, 743–753 (2008).

4. Gepner, Y. *et al.* Effect of Distinct Lifestyle Interventions on Mobilization of Fat Storage Pools: The CENTRAL MRI Randomized Controlled Trial. *Circulation* CIRCULATIONAHA.117.030501 (2017) doi:10.1161/CIRCULATIONAHA.117.030501.

5. Thomas, E. L., Fitzpatrick, J. A., Malik, S. J., Taylor-Robinson, S. D. & Bell, J. D. Whole body fat: Content and distribution. *Progress in Nuclear Magnetic Resonance Spectroscopy* vol. 73 56–80 Preprint at https://doi.org/10.1016/j.pnmrs.2013.04.001 (2013).

6. Gepner, Y. *et al.* Effect of distinct lifestyle interventions on mobilization of fat storage pools CENTRAL magnetic resonance imaging randomized controlled trial. *Circulation* **137**, 1143–1157 (2018).
